# Ambulatory bilateral same‐session vs. unilateral ureteroscopy: A multicentre cohort study

**DOI:** 10.1002/bco2.70109

**Published:** 2026-10-20

**Authors:** Mohamed Abdelkareem, Tawiz Gul, Mohannad Natheef AbuHaweeleh, Humam Emad Rajha, Hamzah Shehadeh, Mohammed Ebrahim, Ibrahim Alnadhari, Osama Abdeljaleel, Abdullah Al‐Ansari, Khaled Al‐Rumaihi, Khalid Al Jalham, Asad Zeidan, Morshed Salah

**Affiliations:** ^1^ Urology Section, Surgery Department, Hazm Mebaireek General Hospital Hamad Medical Corporation Doha Qatar; ^2^ Urology Section, Surgery Department, Ambulatory Care Center Hamad Medical Corporation Doha Qatar; ^3^ Urology Section, Surgery Department, Al Wakra Hospital Hamad Medical Corporation Al Wakra Qatar; ^4^ College of Medicine, QU Health Qatar University Doha Qatar

**Keywords:** bilateral, complications, flexible ureteroscopy, renal stone, stone‐free rate

## Abstract

**Objective:**

To evaluate the safety, efficacy and feasibility of ambulatory bilateral same‐session retrograde intrarenal surgery (BSS‐RIRS) compared to unilateral RIRS, under a standardized clinical protocol.

**Patients and Methods:**

We conducted a **retrospective multicentre cohort study** including 202 adult patients who underwent ambulatory bilateral same‐session RIRS (BSS‐RIRS) or unilateral RIRS between **January 1 and December 31, 2024**. All included patients had complete perioperative data and postoperative non‐contrast CT imaging at 4–6 weeks. Of these, 95 underwent BSS‐RIRS and 107 underwent unilateral RIRS. Demographic, clinical and operative variables were analysed. Stone‐free status (defined as residual fragments ≤4 mm) and complication rates (within 30 days) were assessed. **Multivariable logistic regression** was used to adjust for potential confounders.

**Results:**

BSS‐RIRS patients had significantly longer operative times compared to the unilateral group (106.9 vs. 87.3 minutes, p < 0.001) and were more likely to be treated with thulium lasers (73.4% vs. 54.2%, p = 0.003). Preoperative stenting was notably less common in the BSS‐RIRS group (39% vs. 65.4%, p < 0.001). Despite these differences, early complication rates remained low and statistically similar between groups (2.1% vs. 3.7%, p = 0.49), with adjusted regression analysis showing a possible non‐inferior BSS‐RIRS early complication rate (OR 0.36, p = 0.209). Overall stone‐free rates were comparable (85.3% vs. 82.3%, p = 0.56). In regression analysis, bilateral RIRS showed increased odds of stone‐free status, but this was not statistically supported (adjusted OR 1.62, 95% CI 0.64–4.10, p = 0.309). Grade A stone‐free status (0 mm residual) was significantly lower in the bilateral group (72.8% vs. 93.2%, p < 0.001).

**Conclusion:**

Ambulatory BSS‐RIRS is a safe and effective approach for managing bilateral renal stones, offering comparable overall stone‐free rates and complication profiles to unilateral RIRS. Additionally, it provides procedural efficiency and potential cost‐effectiveness when applied in appropriately selected cases. Further studies are warranted to refine patient selection criteria and validate long‐term outcomes.

## 1 INTRODUCTION

Urolithiasis represents a significant global health concern, with varying incidence and prevalence rates reported worldwide. The disease imposes a considerable clinical and socioeconomic burden, particularly due to its high recurrence rate—approaching 50% within ten years of an initial episode.^1^

The underlying pathophysiology involves a complex interplay of genetic, metabolic and environmental factors. A key contributor is the imbalance between lithogenic substances (e.g., calcium, oxalate) and inhibitors of crystallization (e.g., citrate, magnesium), promoting stone nucleation and growth.^2^

Management strategies for renal calculi span conservative to surgical options, depending on stone size, location, symptomatology and patient‐specific factors. Minimally invasive techniques, especially retrograde intrarenal surgery (RIRS), have gained widespread adoption due to their high efficacy and favourable safety profile. This evolution is fuelled by technological advancements in flexible ureteroscopy—improved optics, greater deflection and smaller scopes—and refinements in laser lithotripsy systems.^3^, ^4^

Traditionally, unilateral RIRS has been the standard for managing single‐sided stone disease. However, the optimal management of bilateral nephrolithiasis remains debated. While some advocate for staged unilateral procedures, others support same‐session bilateral RIRS (BSS‐RIRS) to minimize cumulative operative time, costs and recovery periods. Despite these potential benefits, concerns remain regarding perioperative safety and comparative efficacy and existing studies show significant heterogeneity in design and endpoints.^5^

Importantly, limited data exist on the safety and feasibility of BSS‐RIRS performed in ambulatory (same‐day discharge) settings under standardized protocols. This study aims to evaluate the safety, efficacy and procedural outcomes of ambulatory BSS‐RIRS compared to unilateral RIRS, providing contemporary evidence to guide decision‐making in bilateral stone management.

## 2 MATERIALS AND METHODS

Following ethical approval from our research institution (MRC‐01‐20‐569), we conducted a retrospective analysis of patients who underwent retrograde intrarenal surgery (RIRS) between January 2024 and December 2024 across three hospitals within our corporate network.

### 2.1 Patient Selection

This study included adult patients (≥18 years) who underwent bilateral same‐session or unilateral retrograde intrarenal surgery (RIRS) between **January 1 and December 31, 2024**. Inclusion required complete perioperative data and a mandatory follow‐up non‐contrast computed tomography (NCCT) scan performed within 4–6 weeks postoperatively. Exclusion criteria included absence of NCCT imaging, incomplete medical records, active or recent urinary tract infection (within 6 months) or any known anatomical abnormalities of the urinary tract (e.g., horseshoe kidney, ureteral stricture or duplex system).

A total of 150 patients initially underwent BSS‐RIRS, and 900 underwent unilateral RIRS during the study period. After applying these criteria, 95 bilateral and 107 unilateral cases were eligible for inclusion.

Demographic and clinical data were retrieved from medical records, including age, gender, body mass index (BMI), comorbidities, history of nephrolithiasis, serum creatinine levels, emergency room (ER) visits and use of blood thinners. Operative variables included preoperative stenting, stent type (with or without thread), stent duration, anaesthesia type, operative time, surgeon level of experience and operation‐to‐discharge time. Intraoperative details such as the use of rigid ureteroscopy, access sheath size (10/12, 11/13 or 12/14), laser type (holmium or thulium), lithotripsy techniques (dusting, fragmentation or popcorn), use of Dormia baskets and the need for second‐look procedures were also recorded.

Preoperative stenting was performed based on clinical indications, such as obstructive uropathy with infection or when emergency intervention was required while definitive urine culture results were not yet available. In cases with bilateral renal stones and planned bilateral RIRS, bilateral stenting was occasionally used to facilitate access and improve future procedural outcomes.

All patients underwent preoperative history‐taking, physical examination, laboratory testing and NCCT imaging. Surgery was performed under spinal or general anaesthesia after confirmation of sterile urine cultures. All patients received a single intravenous dose of prophylactic antibiotics at the time of anaesthesia induction, as per the protocol of the participating centre.

A standard surgical protocol was followed, beginning with cystoscopy using a 19–22 Fr sheath and a 30‐degree lens. Pre‐stented patients had stents removed over a guidewire under fluoroscopic guidance. Semirigid ureteroscopy was used to assess the ureter, followed by the insertion of a safety guidewire. A ureteral access sheath (UAS) was then placed, with size dependent on prior stenting status. Flexible ureteroscopy (f‐URS) was performed using Ho:YAG or TFL for lithotripsy. Lower pole stones were repositioned when feasible, and stone fragments were extracted using Nitinol baskets. For bilateral procedures, the same sequence was repeated on the contralateral side if no complications were encountered at the first side of the intervention. At the end of each procedure, bilateral double J stents were inserted, with or without a pull string, at the surgeon's discretion.

Unilateral RIRS followed the same preoperative, intraoperative and postoperative protocol. Patients were observed postoperatively for at least 3 hours and considered eligible for same‐day discharge if they demonstrated stable vital signs, adequate pain control with oral medications, no haematuria, tolerance of oral intake, independent ambulation and the ability to void spontaneously after Foley catheter removal. Those not meeting these criteria were admitted for overnight observation.

Immediate postoperative data, encompassing operative duration and hospitalization duration, was extracted from patient records. Subsequent postoperative assessment was performed by the primary urologist. Radiological outcomes were assessed using follow‐up non‐contrast computed tomography (NCCT) of the kidney, ureter and bladder, performed 4–6 weeks postoperatively. Extracted parameters included stone density (Hounsfield Units), stone number and presence of hydronephrosis. Patients who missed follow‐up or underwent alternative imaging (KUB X‐ray or renal ultrasound) were excluded to ensure consistent and accurate assessment of stone‐free status using NCCT.

Surgical safety was evaluated based on early complications, defined as those occurring within the 30‐day postoperative period. This analysis does not encompass late complications, as systematic follow‐up data beyond 30 days was not available.

Efficacy was assessed using residual fragment outcomes that were defined using a standardized CT‐based classification system. Fragments ≤4 mm were considered **clinically insignificant residual fragments (CIRFs)** and categorized as follows:

- **Grade A**: no residual fragments (0 mm)
- **Grade B**: <2 mm
- **Grade C**: 2–4 mm
- **>4 mm**: considered clinically significant and requiring further evaluation/intervention

## 3 STATISTICAL ANALYSIS

Continuous variables were reported as medians and interquartile ranges (IQRs), while categorical variables were presented as counts and percentages. In line with BJUI‐endorsed guidelines.^6^ We assumed non‐normality for continuous variables and did not perform formal normality testing. Group comparisons were conducted using the Mann–Whitney U test for continuous variables and Chi‐square or Fisher's exact tests for categorical variables, as appropriate.

Multivariable logistic regression was performed to assess associations between surgical type (bilateral vs. unilateral RIRS) and binary outcomes. Covariates included in the logistic regression models were selected based on clinical relevance and observed between‐group imbalances. The models were adjusted for age, gender, BMI, history of nephrolithiasis, comorbidities, stone number, stone density (HU), stone fragmentation, operation duration and preoperative stenting. Additional models also adjusted for stone dusting technique and dilation status where appropriate.

## 4 RESULTS

### 4.1 Study Population and Demographics

The study cohort comprised 202 patients undergoing RIRS, with 95 patients undergoing bilateral same‐session RIRS and 107 patients undergoing unilateral RIRS procedures. Comparative analysis revealed no significant differences in baseline characteristics between groups. The median age was similar (37 vs. 40 years, p = 0.079), as was gender distribution (94.7% vs. 99.1% male, p = 0.07), median BMI (26.67 vs. 26.35 kg/m^2^, p = 0.90) and prevalence of comorbidities (39.4% vs. 38.3%, p = 0.88). Both groups demonstrated equivalent postoperative recovery times, with identical median operation‐to‐discharge intervals of 6 hours (p = 0.64). Further details are reported in Table 1.

**TABLE 1:** Demographic characteristics of patients who underwent bilateral same session RIRS compared to unilateral RIRS.

Variable		Bilateral Same Session RIRS*	Unilateral RIRS*	
**N**		95	107	
**Age, median (IQR)**		37 (31, 46)	40 (35, 43)	p = 0.079
**Gender, n (%)**	Male	90 (94.7%)	106 (99.1%)	p = 0.07
	Female	5 (5.3%)	1 (0.9%)	
**BMI, median (IQR)**		26.67 (24, 29.36)	26.35 (24.02, 29.36)	p = 0.90
**Comorbidities** ^**#**^ **, n (%)**	Yes	37 (39.4%)	41 (38.3%)	p = 0.88
	No	57 (60.6%)	66 (61.7%)	
**History of stone disease, n (%)**	Yes	63 (66.3%)	58 (54.2%)	p = 0.08
	No	32 (33.7%)	49 (45.8%)	
**Serum creatinine, median (IQR)**		89 (76, 101)	85 (77, 93)	p = 0.19
**ER visit, n (%)**	Yes	82 (86.3%)	99 (92.5%)	p = 0.15
	No	13 (13.7%)	8 (7.5%)	
**Blood thinners, n (%)**	Yes	0 (0.0%)	2 (1.9%)	p = 0.18
	No	95 (100.0%)	105 (98.1%)	

* RIRS: Retrograde intrarenal surgery.

^#^ Comorbidities refer to chronic medical conditions including, diabetes mellitus, hypertension, cardiovascular disease and chronic kidney disease.

### 4.2 Comparative Clinical and Operative Outcomes

Table 2 describes the clinical and operative results across patients undergoing bilateral and unilateral retrograde intrarenal surgery (SS‐RIRS). The bilateral SS‐RIRS cohort exhibited distinct clinical features, including a higher, though not statistically significant, prevalence of stone disease history. (66.3% vs. 54.2% p = 0.08) and marginally higher serum creatinine levels (89 vs. 85 μmol/l, p = 0.19). This group presented more frequently with haematuria (5.3% vs. 2.8%, p = 0.37), febrile symptoms (2.1% vs. 0%, p = 0.13) and lower urinary tract symptoms (4.2% vs. 1.9%, p = 0.33). Conversely, they demonstrated reduced rates of emergency department presentations (86.3% vs. 92.5%, p = 0.15) and preoperative stent placement (39% vs. 65.4%, p < 0.001).

**TABLE 2:** Preoperative, Operative and Postoperative attributes of Bilateral Same Session RIRS compared to Unilateral RIRS.

Variable		Bilateral Same Session RIRS*	Unilateral RIRS*	
**N**		95	107	
**Preoperative stenting, n (%)**	Yes	37 (39.0%)	70 (65.4%)	p < 0.001
	No	58 (61.0%)	37 (34.6%)	
**Double J stent type**	With thread	59 (66.3%)	66 (61.6%)	p = 0.59
	Without thread	30 (33.7%)	40 (37.4%)	
**Double J stent duration (days), median (IQR)**		6 (5, 14)	6 (5, 14)	p = 0.38
**Operation duration (minutes), median (IQR)**		105 (80, 125)	80 (62, 110)	p < 0.001
**Surgeon level of experience, n (%)**	Resident	2 (2.1%)	3 (2.8%)	p = 0.26
	Fellow	4 (4.2%)	1 (0.9%)	
	Specialist	40 (42.1%)	56 (52.3%)	
	Consultant	49 (51.6%)	47 (43.9%)	
**Operation to discharge time (hours), median (IQR)**		6 (5, 7.75)	6 (5, 7)	p = 0.64
**Anaesthesia type, n (%)**	General	4 (4.2%)	15 (14.9%)	p = 0.035
	Spinal	91 (95.8%)	91 (85.1%)	
**Rigid ureteroscopy, n (%)**	Yes	78 (87.6%)	105 (99.1%)	p < 0.001
	No	11 (12.4%)	1 (0.94%)	
**Sheath size, n (%)**	10/12	36 (41.4%)	50 (51.6%)	p = 0.19
	11/13	39 (44.8%)	42 (43.3%)	
	12/14	10 (11.5%)	4 (4.1%)	
**Type of laser, n (%)**	Holmium	20 (21.3%)	47 (43.9%)	p = 0.003
	Thulium	69 (73.4%)	58 (54.2%)	
**Stone dusting, n (%)**	Yes	76 (85.4%)	88 (89.8%)	p = 0.36
	No	13 (14.6%)	10 (10.2%)	
**Stone fragmentation, n (%)**	Yes	37 (40.7%)	27 (27.6%)	p = 0.057
	No	54 (59.3%)	71 (72.4%)	
**Popcorn lithotripsy, n (%)**	Yes	42 (47.2%)	27 (27.6%)	p = 0.005
	No	47 (52.8%)	71 (72.4%)	
**Dormia used, n (%)**	Yes	41 (44.6%)	40 (37.4%)	p = 0.30
	No	51 (55.4%)	67 (62.6%)	
**Second look operation, n (%)**	Yes	6 (6.4%)	9 (8.4%)	p = 0.61
	No	88 (93.6%)	98 (91.6%)	

* RIRS: Retrograde intrarenal surgery.

Bilateral procedures required significantly longer median operative durations (105 vs. 80 minutes, p < 0.001) and showed preferential utilization of Thulium laser technology (73.4% vs. 54.2%, p = 0.003). The popcorn lithotripsy technique was employed more frequently in bilateral cases (47.2% vs. 27.6%, p = 0.005), while unilateral procedures demonstrated greater use of Holmium laser systems (21.3% vs. 43.9%, p = 0.003) and a non‐significant trend towards increased stone fragmentation (40.7% vs. 27.6%, p = 0.057). Clinical outcomes were equivalent between groups, with no statistically significant differences observed in the requirement for secondary procedures (6.4% vs. 8.4%, p = 0.61) or rates of same‐day discharge (95.8% vs. 95.3%, p = 0.87).

Second‐look interventions were required in 6.4% and 8.4% of unilateral and bilateral SSRIRS cases, respectively, as shown in Figure 1.

**FIGURE 1:**
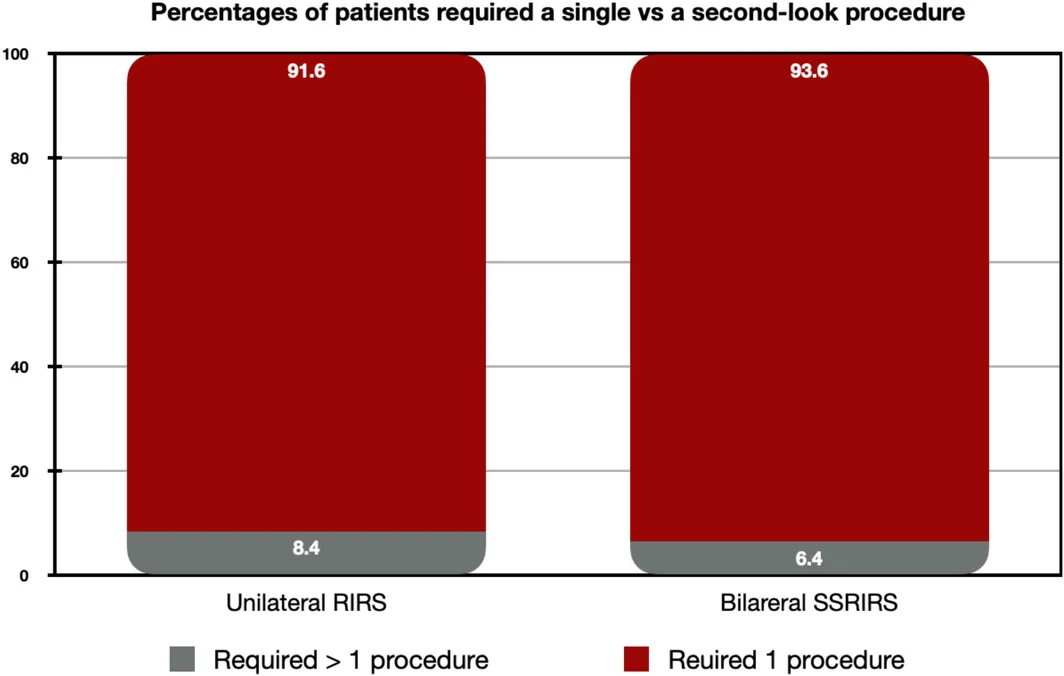
Percentages of patients required a single vs a second‐look procedure.

### 4.3 Stone Metrics and Radiological Characteristics

The stone metrics and radiological characteristics were reported from the right and left sides of patients undergoing bilateral SS‐RIRS as well as those undergoing unilateral SS‐RIRS, reported in Table 3. Patients undergoing bilateral same‐session RIRS demonstrated significantly lower stone sizes and density compared to unilateral procedures. The median stone sizes were 650 (IQR: 432, 1269) and 830 (IQR: 582, 1200) for right and left‐sided stones in patients with bilateral stones, and 1040.15 (IQR: 678, 1960.352) for patients with unilateral stones. The median Hounsfield Units (HU) values were 950 (IQR: 700, 1200) for right‐sided stones and 1000 (IQR: 700, 1300) for left‐sided stones in the bilateral group, versus 1100 (900, 1300) in unilateral cases. On the other hand, the proportion of single versus multiple stones did not differ significantly between groups. In bilateral RIRS, single stones were present in 41.5% (right) and 38.3% (left) of cases, compared to 44.9% in unilateral procedures (right vs. unilateral: p = 0.63; left vs. unilateral: p = 0.53). Multiple stones were observed in 58.5% (right) and 61.7% (left) of bilateral cases versus 55.1% of unilateral cases. Dilatation was present in 57.1% (right) and 59.3% (left) of bilateral RIRS patients, compared to 63.2% of unilateral cases. These differences were not statistically significant (right vs. unilateral: p = 0.39; left vs. unilateral: p = 0.58).

**TABLE 3:** Stone metrics comparison by side of Bilateral Same Session RIRS compared to Unilateral RIRS.

Variable		Bilateral Same Session RIRS^#^		Unilateral RIRS^#^	*	**
		Right	Left			
**N**		95	95	107		
**Stone size (mm** ^**3**^ **), median (IQR)**		650 (432, 1269)	830 (582, 1200)	1040.15 (678, 1960.352)	p < 0.001	p = 0.006
**Hounsfield Units, median (IQR)**		950 (700, 1200)	1000 (700, 1300)	1100 (900, 1300)	p = 0.027	p = 0.036
**Stones number, n (%)**	Single	39 (41.5%)	36 (38.3%)	48 (44.9%)	p = 0.63	p = 0.53
	Multiple	55 (58.5%)	58 (61.7%)	59 (55.1%)		
**Dilatation, n (%)**	Yes	52 (57.1%)	54 (59.3%)	67 (63.2%)	p = 0.39	p = 0.58
	No	39 (42.9%)	37 (40.7%)	39 (36.8%)		

^#^ RIRS: Retrograde intrarenal surgery.

* Difference between Right Bilateral Same Session RIRS and Unilateral Same Session RIRS.

** Difference between Left Bilateral Same Session RIRS and Unilateral Same Session RIRS.

### 4.4 Outcomes of Safety and Efficacy

Safety was assessed based on early and late complications, while efficacy was evaluated according to stone‐free status and stone‐free grade, as summarized in Table 4. The rates of early complications did not differ significantly between bilateral and unilateral RIRS (4.2% vs. 8.4%, p = 0.22), suggesting comparable safety profiles between the two approaches. CIRFs (defined as ≤4 mm residual) were achieved in 85.3% of bilateral and 82.3% of unilateral cases (p = 0.56). However, stone‐free grades differed significantly between groups: bilateral RIRS demonstrated lower Grade A (0 mm/no residual) (72.8% vs. 93.2%, p < 0.001) but higher Grade B (<2 mm residual) (7.4% vs. 0.0%) and Grade C (2–4 mm residual) (19.8% vs. 6.8%) compared to unilateral RIRS. Notably, bilateral RIRS was associated with lower rates of significant residual stones (>4 mm, classified as “not stone‐free”) (14.7% vs. 17.7%, p = 0.56), though this difference was not statistically significant. Figure 2 depicts a visual presentation of stone‐free status across bilateral and unilateral SS‐RIRS.

**TABLE 4:** Outcomes of Efficacy and Safety in patients undergoing Bilateral Same Session RIRS compared to Unilateral RIRS.

Variable		Bilateral Same Session RIRS*	Unilateral Same Session RIRS*	
**N**		95	107	
Early complications**, n (%)	Yes	4 (4.2%)	9 (8.4%)	p = 0.22
	No	91 (95.8%)	98 (91.6%)	
CIRFs*** n (%)	Yes	81 (85.3%)	88 (82.3%)	p = 0.56
	No^§^	14 (14.7%)	19 (17.7%)	
**CIRFs subcategories, n (%)**	Grade A	59 (72.8%)	82 (93.2%)	p < 0.001
	Grade B	6 (7.4%)	0 (0.0%)	
	Grade C	16 (19.8%)	6 (6.8%)	

* RIRS: Retrograde intrarenal surgery. ** Early complications within 30 days. *** Clinically Insignificant Residual Fragments. ^ Stone‐free grade details are as follows: Grade A: 0 mm/no residual on either side, Grade B: <2 mm residual on either side, Grade C: 2–4 mm residual on either side. § Not stone free: >4 mm residual on either side.

**FIGURE 2:**
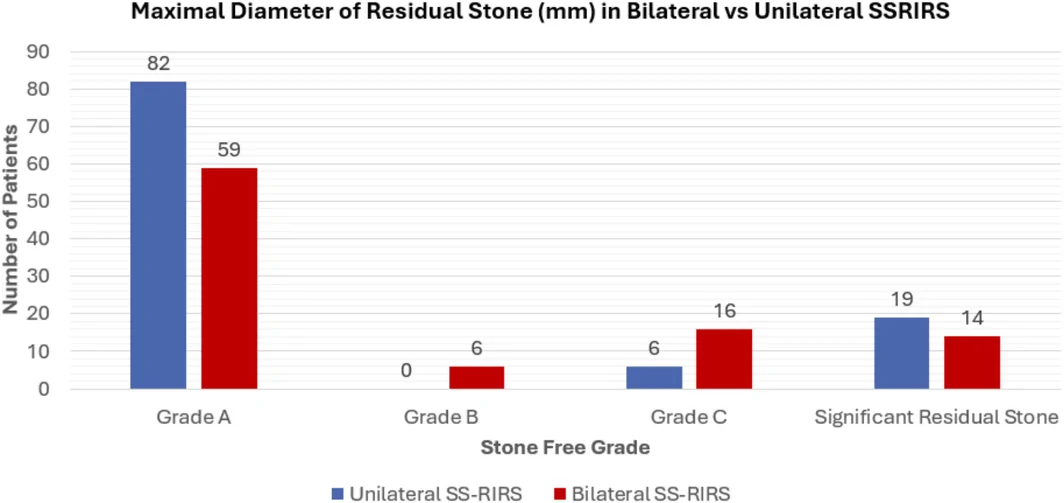
Maximal Diameter of Residual Stone (mm) in Bilateral vs Unilateral SSRIRS.

### 4.5 Multivariable Analysis of Surgery Type and Efficacy and Safety Outcomes

Adjusted logistic regression analysis, illustrated in Table 5, early complications analysis showed that bilateral RIRS demonstrated 64% lower odds (OR 0.36, 95% CI 0.07–1.78, p = 0.209), with weak evidence against the model hypothesis. This suggests that ambulatory bilateral RIRS is possibly not inferior to unilateral RIRS in postoperative complications, but the wide confidence intervals crossing unity indicate a lack of statistical precision and preclude definitive conclusions. Regarding stone‐free status, bilateral RIRS showed a 1.62‐fold increase in odds (OR 1.62, 95% CI 0.64–4.10, p = 0.309) of achieving stone‐free status, with weak evidence against the model hypothesis.

**TABLE 5:** Logistic regression between Surgery type and Safety and Efficacy outcomes comparing Bilateral Same Session RIRS to Unilateral Same Session RIRS.

Outcome†		OR	P‐value	Lower CI	Upper CI
Early Complications*	Raw	0.48	0.233	0.14	1.61
	Adjusted**	0.36	0.209	0.07	1.78
**Stone Free**	Raw	1.25	0.563	0.59	2.65
	Adjusted^#^	1.62	0.309	0.64	4.10

^†^ Unilateral Same Session RIRS group is used as reference. * Early complications within 30 days.

** Model adjusted for age, gender, BMI, nephrolithiasis history, comorbidities, stone fragmentation, stone number, stone density (HU), operation duration and preoperative stenting. # Model additionally adjusted for stone dusting and dilation.

## 5 DISCUSSION

With advancements in flexible ureteroscopic technology, retrograde intrarenal surgery (RIRS) has expanded beyond traditional guidelines for stone size (<1.5 cm), especially in high‐volume centres where it demonstrates favourable outcomes compared to more invasive procedures like percutaneous nephrolithotomy (PCNL) and shockwave lithotripsy (SWL). These benefits include shorter hospital stays, fewer complications and acceptable clearance outcomes.^3^, ^4^, ^7^

Among these developments, bilateral same‐session RIRS (BSS‐RIRS) has gained attention for treating bilateral renal stones in a single procedure. While guidelines remain limited, multiple studies report CIRF (clinically insignificant residual fragment) rates ranging from 73% to 90.5%, with most complications being Clavien‐Dindo grade ≤2.^5^, ^8^, ^9^, ^10^, ^11^, ^12^ More recently, innovations like suction‐based access sheaths have enhanced clearance rates and reduced complication risks further.^13^, ^14^

In our cohort, the incidence of early complications, including febrile UTI, did not differ significantly between the bilateral and unilateral groups. Notably, two patients (2.1%) in the bilateral RIRS group experienced febrile UTI, whereas no such events occurred in the unilateral group (p = 0.13). However, this difference did not reach statistical significance. Adjusted logistic regression analysis similarly confirmed the absence of a significant difference in overall early complications, demonstrating that bilateral RIRS was associated with 64% lower odds of early adverse events (adjusted OR 0.36, 95% CI 0.07–1.78, p = 0.209). Nonetheless, the wide confidence interval crossing unity underscores a lack of statistical precision, thereby precluding definitive conclusions regarding non‐inferiority.

Our findings are consistent with a recent meta‐analysis by Lepine et al.,^15^ which found that bilateral RIRS carries higher retreatment and complication rates in select subgroups (e.g., elderly), but maintains comparable overall CIRF outcomes. Differences in methodology, such as our use of CT‐based stratification, strict exclusion of infected or anatomically complex cases and an ambulatory surgery protocol, may explain our lower complication rates.

Regarding stone clearance, CIRF rates were comparable between groups (85.3% vs. 82.3%), but Grade A outcomes (0 mm residual fragments) were significantly more common in the unilateral group (93.2% vs. 72.8%, p < 0.001). This may reflect the higher prevalence of preoperative stenting in the unilateral arm (65.4% vs. 39%, p < 0.001), which is known to facilitate access and potentially improve complete stone clearance. While our findings align with the FLEXOR study,^16^ which reported no significant difference in overall CIRFs with or without pre‐stenting, we observed improved Grade A outcomes in pre‐stented cases. This distinction is supported by a recent meta‐analysis by Yuen et al. (2025), which demonstrated that pre‐stenting significantly reduces access failure rates (RR ≈ 0.44), shortens operative time and enhances stone‐free outcomes.^17^ Similarly, Ansari et al. (2025) reported higher complete clearance rates (85.3% vs. 64.7%; p = 0.007) and shorter operative durations in pre‐stented patients.^18^ These findings suggest that while pre‐stenting may not impact overall CIRFs, it appears to enhance the quality of clearance, particularly when aiming for complete (Grade A) stone‐free status.

Our results also support the feasibility of ambulatory bilateral RIRS, with a median operation‐to‐discharge time of 6 hours. This is consistent with Castellani et al.,^19^ who reported that 14% of global centres perform same‐day discharges. Other studies also confirmed no significant differences in complications or clearance between unilateral and bilateral RIRS, further supporting its safety.^20^

Importantly, Danilovic et al.^5^ found equivalent outcomes between both kidneys in bilateral RIRS cases, echoing our own findings. Castellani et al.^12^ and Corrales et al.^21^ also showed favourable safety profiles, though their reported sepsis rates (11.1%) highlight the need for rigorous patient selection.

Stone burden remains critical for surgical planning. Our study supports previous work by Alkan et al.^8^ and Huang et al.,^22^ who found higher CIRF rates in patients with stone burdens <2–2.5 cm. Careful selection of patients with moderate stone burden is therefore essential.

BSS‐RIRS offers additional benefits in cost‐efficiency. Though a formal cost analysis was beyond this study's scope, prior research indicates that single‐session bilateral interventions reduce hospital resource use by 15–30% compared to staged approaches.^10^, ^11^, ^19^

Finally, we acknowledge the limitations of this study. Its retrospective design, relatively limited sample size and variability in institutional protocols may introduce bias. The absence of rigid stone burden criteria in the unilateral group may also affect the generalizability of our findings. Moreover, the male predominance in our cohort limits the applicability of the findings to more gender‐diverse populations. However, our outcomes were comparable to those reported in studies with more gender‐diverse cohorts. Furthermore, our safety analysis was limited to early complications (within 30 days); data on late complications were not systematically collected, which precludes analysis of longer‐term outcomes. Prospective, multicentre studies with broader demographic representation are warranted to validate these results.

## 6 CONCLUSION

Ambulatory bilateral same‐session ureterorenoscopy (BSS‐RIRS) demonstrates safety and efficacy comparable to unilateral RIRS in appropriately selected patients. Despite a lower proportion of Grade A stone‐free outcomes in the bilateral group, overall stone‐free rates (≤4 mm residual) and complication rates were similar between groups. The bilateral approach offers procedural efficiency and potential cost advantages without compromising clinical outcomes. Preoperative stenting and total stone burden may influence success, and further prospective studies are warranted to optimize selection criteria.

## AUTHOR CONTRIBUTIONS

T.G. and M.A. contributed equally to this work and share first authorship.

## CONFLICT OF INTEREST STATEMENT

The authors declare that they have no conflicts of interest.

## Supporting information


**Supplementary Table S1.** Early (≤30‐day) postoperative complications, categorized according to the modified Clavien–Dindo classification and corresponding management.
