# Supplementary material for: Ambulatory bilateral same‐session vs. unilateral ureteroscopy: A multicentre cohort study
Source: BJUI Compass. 2026 Oct 20;7(10):e70109. doi: 10.1002/bco2.70109 (PMC13681178; doi:10.1002/bco2.70109)
Supplement: Supplementary file 1 [file BCO2-7-e70109-s001.docx]

**Supplementary Table S1. Early (≤30-day) postoperative complications, categorized according to the modified Clavien–Dindo classification and corresponding management.**

| Complication | Surgery | Number of Patients | Admissions | Management | Clavien–Dindo classification |
| --- | --- | --- | --- | --- | --- |
| Fever | Bilateral RIRS | 2 | 2 | Admitted for IV antibiotic | 2 |
|  | Unilateral RIRS | 0 | 0 |  |  |
| Hematuria | Bilateral RIRS | 5 | 0 | Reassurance and discharge from ER | 1 |
|  | Unilateral RIRS | 3 | 0 |  |  |
| Flank Pain | Bilateral RIRS | 12 | 2* | Reassurance and discharge from ER | 1 |
|  | Unilateral RIRS | 8 | 0 |  |  |
| Lower Urinary Tract Symptoms | Bilateral RIRS | 4 | 0 | Treated conservatively | 1 |
|  | Unilateral RIRS | 2 | 0 |  |  |

* Same patient had flank pain and fever, a total of 2 patients where admitted
